# Red Light Stimulates Feeding Motivation in Fish but Does Not Improve Growth

**DOI:** 10.1371/journal.pone.0059134

**Published:** 2013-03-14

**Authors:** Gilson L. Volpato, Thais S. Bovi, Renato H. A. de Freitas, Danielle F. da Silva, Helton C. Delicio, Percilia C. Giaquinto, Rodrigo Egydio Barreto

**Affiliations:** 1 Department of Physiology, Bioscience Institute, Caunesp, São Paulo State University (UNESP), Botucatu, SP, Brazil; 2 Department of Morphology, Laboratory of Fish Biology and Genetics, Bioscience Institute, São Paulo State University (UNESP), Botucatu, SP, Brazil; University of Hamburg, Germany

## Abstract

Nile tilapia fish were individually reared under similar light levels for 8 weeks under five colored light spectra (maximum wavelength absorbance): white (full light spectrum), blue (∼452 nm), green (∼516 nm), yellow (∼520 nm) or red (∼628 nm). The effects of light on feeding, latency to begin feeding, growth and feed conversion were measured during the last 4 weeks of the study (i.e., after acclimation). We found that red light stimulates feeding, as in humans, most likely by affecting central control centers, but the extra feeding is not converted into growth.

## Introduction

The effects of environmental color on fish have been increasingly studied during this century, particularly after the first publication of endocrine modulation by light color [1]. The reported effects of light color on fish mostly involve production variables, such as growth [2]–[6], feeding [7], reproduction [8], stress [4], [9]–[12] and survival [13].

Here, we investigated the effects of light color on fish growth. Because growth represents a balance between energy obtained and energy demand, we hypothesized that color might affect these two processes. Colors have been shown to produce different effects on fish in a species-specific manner [8], [14]. Therefore, we measured feeding motivation, feed intake and growth under white, blue, yellow, red or green light in the Nile tilapia, *Oreochromis niloticus*, an animal that has a broad range of wavelength sensitivity [15]. When imposing experimental color regimes on fish, researchers simultaneously address the effects of colors on the adjustment of the animal to the novel environment and the effects of colors on the studied parameters (e.g., growth). To avoid this confound, we investigated the chronic effects of environmental light color only after the fish had adjusted to the colored light environment.

## Materials and Methods

### 1. Ethical note

This study met the guidelines of the Brazilian College for Animal Experimentation (COBEA; http://www.cobea.org.br) and was approved by the Ethics Committee for Animal Experimentation (CEEA) of the Bioscience Institute, UNESP, Botucatu, SP, Brazil (Protocol #151-CEEA, August 2009).

### 2. General statements

In the first study we investigated the effects of light color on feeding motivation, feed intake, feed conversion and growth. Considering that red light increased the frequency of fast feeding behavior (<10 s), but this response could be attributed to better visual contrast of the food pellets in the red environment, a second study was performed. We excluded the vision effect by testing the feeding behavior of fish that were kept in a colored environment both during the absence of light (test 1) and in response to chemical-only pellet cues (test 2).

### 3. Animals

A stock population of Nile tilapia (5 fish/L) was maintained in indoor tanks that used a recirculating water system (DO >4.0 mg/L; nitrite and ammonium levels lower than 0.05 and 0.5 mg/L, respectively; pH 6.5; 25°C – 27°C; photophase from 06:00 to 18:00 h). Aeration was supplied through air stones connected to the aquarium air pumps. The fish were fed once a day, 5 days a week, throughout the study (feeding schedule based on a previously described protocol [16]) with commercial tropical fish pellets (Socil Pró-Pecuária S/A) composed of 6% water, 30% crude protein, 8% crude fat, 7% crude fiber and a 9% mineral mixture.

### 4. Study 1

Fish from the stock population were randomly caught and individually maintained for 8 weeks in a glass aquarium (60× 60× 30 cm) under one of five light color conditions (maximum wavelength absorbance): white (full spectrum), blue (∼452 nm), green (∼516 nm), yellow (∼520 nm) or red (∼628 nm), under similar light levels. The fish were fed once a day, 5 days a week, throughout the experiment. Growth, feeding and feed conversion efficiency were measured during the last 4 weeks. The fish were weighed before feeding on the first day of the 5^th^ week (initial) and on the last day of the 8^th^ week (final). The amount of ingested food was measured daily, 5 days per week, and the conversion efficiency during the last 4 weeks was calculated. The weight of the fish was similar among the color treatment groups on the first day of the 5^th^ week (ANOVA, P = 0.36; fish exposed to white light weighed 14.33±2.09 g; to blue, 15.47±0.92 g; to green, 14.87±1.97 g; to yellow, 16.48±2.11 g; and to red, 14.58±2.01 g).

Evaluation of feeding motivation was based on the latency to begin feeding and feed intake. The latency to begin feeding was defined as the timed from the introduction of the pellets into the aquaria (the pellets stayed on the surface) to the first snatch of a pellet. Fast feeding was assessed on 20 consecutive days, and the percentage of fast feeding in this period was calculated.

Feed intake was determined for each feeding day by calculating the difference between the amount of food offered and the amount of food remaining after 20 min. Eighty size-matched food pellets (∼2 mm in diameter totaling 3.5% of individual fish biomass) were given per day. Feed intake was measured in terms of the total number of pellets ingested during the last 4 weeks and was transformed into a food weight/body weight ratio.

Growth was defined by the specific growth rate (SGR), calculated as SGR (%.day^−1^)  =  (LogBW_f_ – LogBW_i_).100/Δt, where BW_f_ is the final body weight, BW_i_ is the initial body weight, and Δt is the days of the experiment after acclimatization (Δt = 28 d). Weight gain was defined as BW_f_ – BW_i_. The feed conversion efficiency (FCE) was calculated as the ratio of weight gain/food ingested.

The aquaria were illuminated with white fluorescent light. As light intensity can influence fish growth {[17], [18], [19]}, different environmental colors were obtained by covering each aquarium with layers of cellophane to achieve similar light intensity (ANOVA, P = 0.20). The white aquaria presented a mean (± sd) light intensity of 47±12 lux, red of 38±9 lux, green of 45±11 lux, blue of 48±9 lux and yellow of 56±18 lux. During the experiment, the mean temperature of the water was similar among treatments (ANOVA, P = 0.22): white, 20.5±2.7°C; red, 21.1±2.7°C; green, 20.9±2.5°C; blue, 20.7±2.6°C; yellow, 21.0±2.6°C. Six fish were assigned to each color treatment, but one fish died in the yellow color condition.

### 5. Study 2

#### 5.1. General Conditions

Fish from the stock population were randomly caught and individually held in glass aquaria (40 cm × 23 cm × 25 cm; ∼21 L of water) under the experimental environmental color conditions for 15 weeks, as specified in study 1. The laboratory was illuminated with white fluorescent light and each aquarium was covered with a different number of cellophane layers to achieve similar light intensity (ANOVA; P = 0.69). The white aquaria presented a mean (± SD) light intensity of 71±7 lux, red of 75±12 lux, green of 75±11 lux, blue of 68±14 lux and yellow of 76±12 lux. The predominant color wavelengths were the same as in Study 1. The water temperature was similar among treatments (ANOVA, P = 0.09): 23.7±0.8°C (red), 23.3±0.9°C (yellow), 23.6±0.7°C (blue), 22.2±1.2°C (white) and 23.2±0.8°C (green).

Fish were weighed on the first day of week 9 and at the end of the study and feeding occurred once a day as previously described. Initial body weight was not different among the fish at the beginning of week 9: fish in white light weighed (mean ± SD) 47.00±8.77 g; in blue, 45.97±8.12 g; in green, 47.37±6.68 g; in yellow, 47.93±9.73 g; and in red, 46.05±4.06 g (ANOVA, P = 0.99). Fish were food-deprived for 24 h before each feeding test. Six fish were tested in each color condition; however, one fish in the white color condition was not tested in test 2.

#### 5.2. Test 1 – Feeding during the absence of light

**Figure 1:**
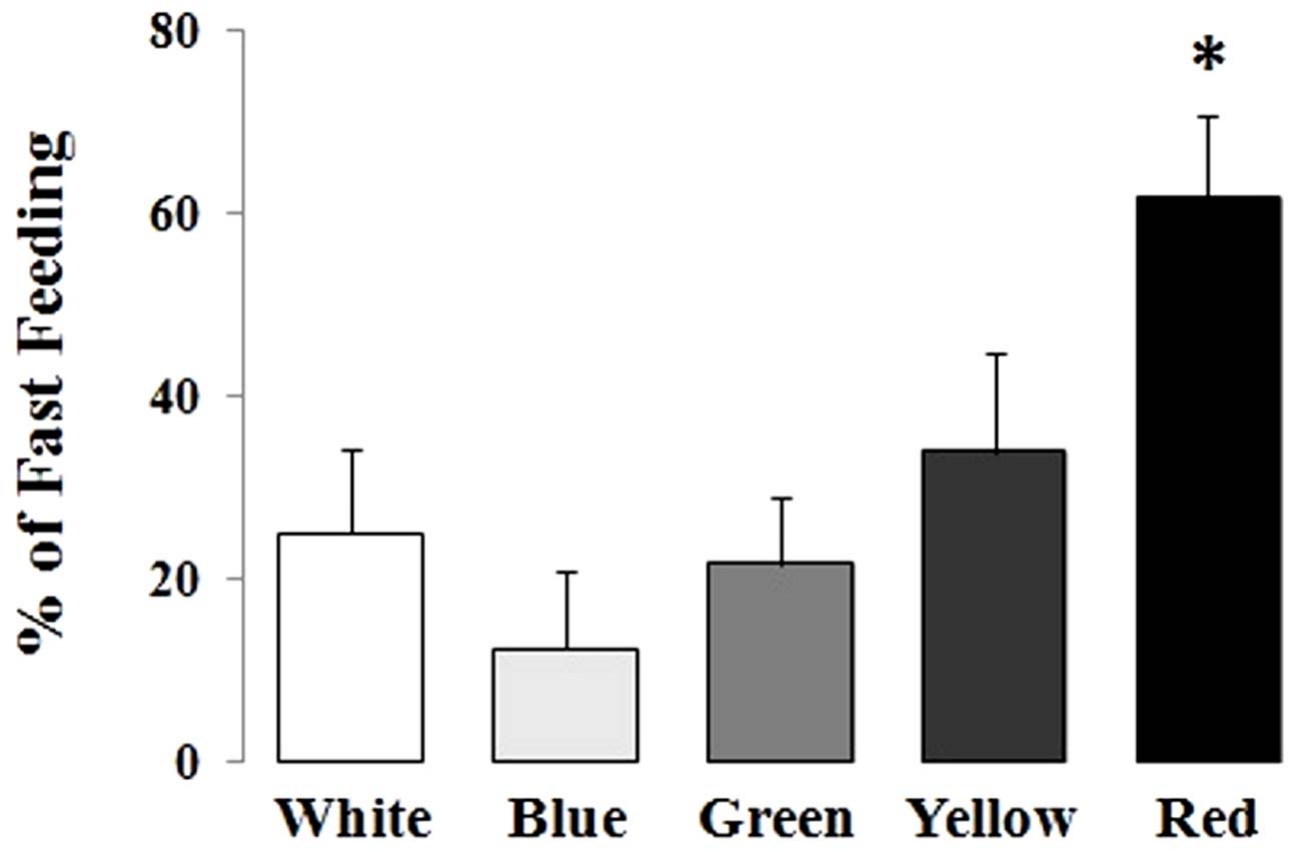
Mean (± SEM) percentage of days fish snatched food pellets within 10 s in each color treatment. *N* = 6, except for the yellow *N* = 5. Asterisk indicates the mean is different from the other groups (ANOVA, P<0.05).

Each fish was tested once a day, on 6 random days between the 9^th^ and 11^th^ weeks of the experiment. Testing occurred from 18:30 h to 19:30 h, in the absence of light, approximately 33 h after the last feeding. In each test, pellets of food were introduced into the aquarium 30 min after the light was turned off. Using video recordings (infrared recordings filmed in darkness), we determined, as a measure of feed motivation, the time elapsed to first snatch after the pellets were placed into the aquarium (latency to first snatch). From these data, we also calculated the frequency (out of 6) of fish that began feeding in less than 10 s (a criteria based on study 1, Fig. 1).

#### 5.3. Test 2 – Feeding behavior induced by chemical cues

This test was carried out between the 13^rd^ and 15^th^ weeks of the study. Each fish was tested once a day on 5 random days in the morning (24 h after the last feeding). Briefly, we introduced a solution of food (chemical cue) into a corner of the aquarium and measured the time that elapsed until the fish approached the chemical cue (corner area).

Aeration ceased 30 min before the test to avoid spreading the chemical cue throughout the aquarium. Next, using a Pasteur pipette, 3 mL of the chemical cue was injected 1 cm above the surface of the water into one upper corner of the aquarium (the fish was always in the opposite side of the aquarium), close to the aquarium wall. An area measuring 15 cm from the releasing area to the opposite side of the aquarium was visually delimited by a vertical line to indicate the feeding area, which included the corner where the food chemical was released. This size was used because it is a little bit greater than the total body length (∼12.5 cm) of the fish. We examined whether each fish entered this feeding area less than 30 s after release of the chemical cue. The 30-s limit was determined by previous tests using food chemical cues colored with methylene blue as the time necessary for the chemical cue to reach the water outside the feeding area. The fish was considered to be inside the feeding area if it crossed the vertical line with the anterior part of its body (head into the feeding area).

The chemical cue (food solution) was prepared by diluting 25 g of feed pellets in 400 mL of water (∼63 mg of pellets/1 ml of water) for 24 h before the test. This solution was filtered (1 mm × 1 mm mesh) and used at the same temperature as the aquarium water.

### 6. Statistics

Parametric assumptions were tested using Levene's (homoscedasticity) and Kolmogorov-Smirnov's (normality) tests. Due to the high number of zero values, we added a constant (0.1) to each value and calculated the square root whenever necessary. Mean values were compared among the color treatments using one-way ANOVAs and post-hoc comparisons with LSD tests. Correlations were performed using Pearson's tests. For all statistics, *α* = 0.05.

## Results

### 1. Motivation for Feeding

In study 1 we found that light color affected motivation for feeding; that is, fast feeding behavior was more frequently observed in the red light condition (Fig. 1).

**Figure 2:**
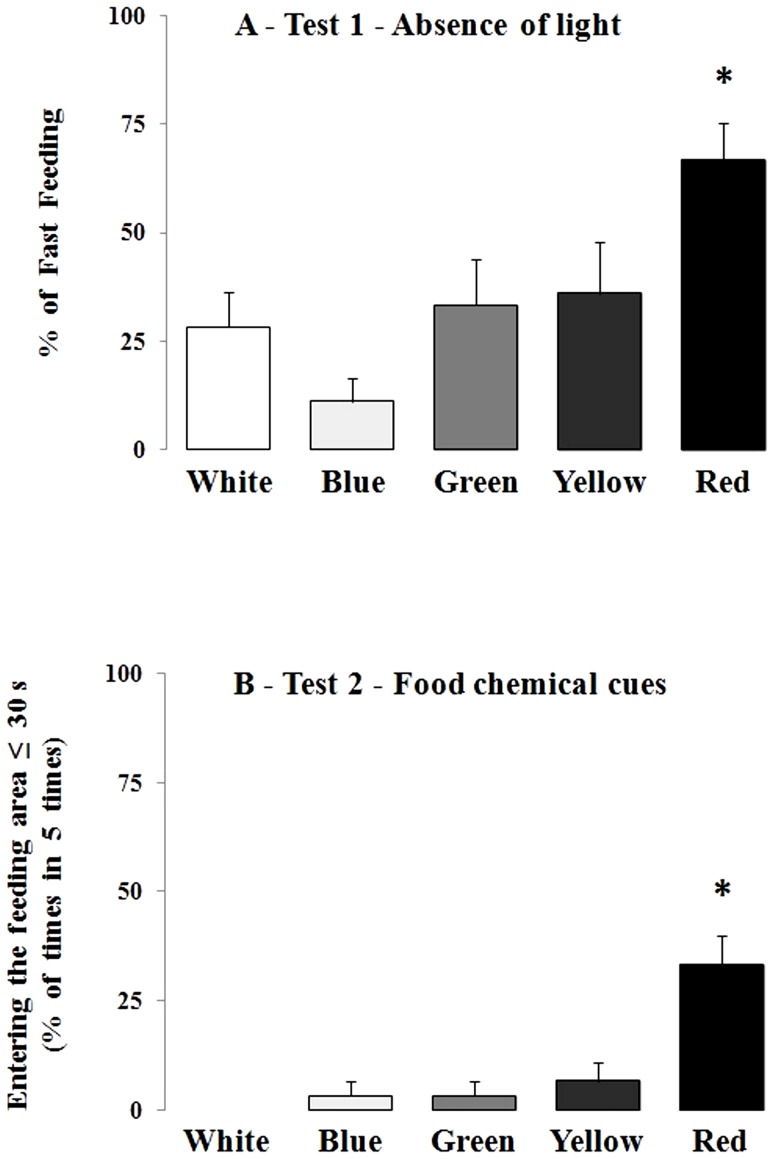
Effect of holding color on feeding behavior stimulated by non-visual cues. A – Percentage of first snatch emitted to pellets in the absence of light. N = 6 fish each color. B – Feeding behavior (entering the chemical cue area) stimulated by food chemical cues (no pellets were present). Means (± SEM) of 6 fish for each color (white color N = 5). Asterisk indicates higher mean compared to the other color treatments (not different among themselves): ANOVA, A) P = 0.005 and B) P = 0.000052.

Study 2 corroborated the effect of light color on feeding motivation suggested in study 1. In test 1, we calculated the percentage of each color-conditioned fish that began feeding in less than 10 s (based on study 1) in the absence of light (5 tests, mean percentage per fish, then the mean percentage for all fish in the same color condition). We found that a higher percentage of fish previously conditioned to the red light began feeding in less than 10 s (fast feeding) compared to fish conditioned to the other colors (Fig. 2A; ANOVA, P = 0.005). In test 2, a higher percentage of fish reared in red light reached the feeding area in less than 30 s compared to the other treatments (Fig. 2B; ANOVA, P = 0.000052).

### 2. Food ingestion and growth

**Figure 3:**
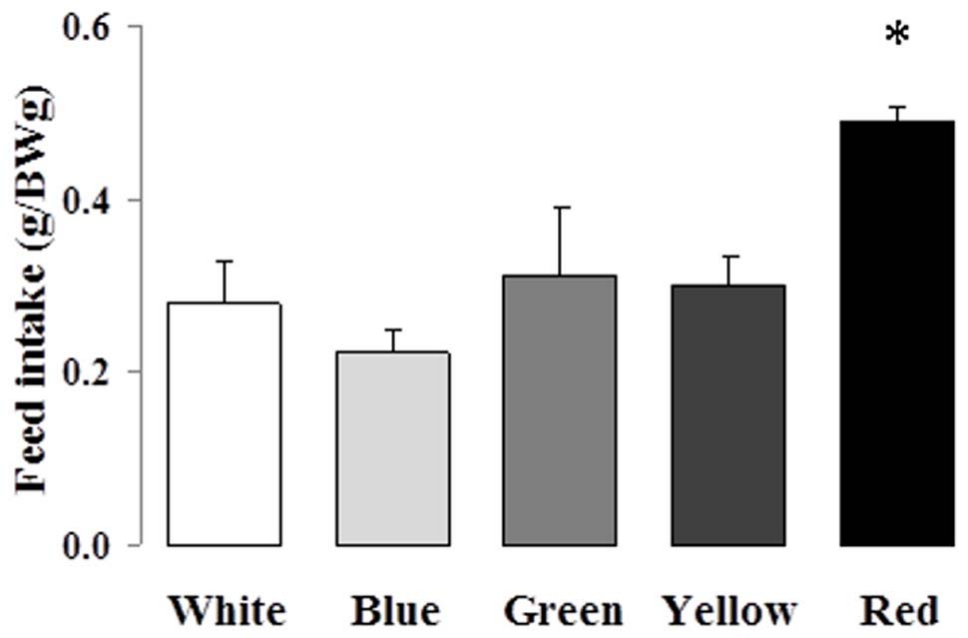
Mean (±SEM) feed intake of Nile tilapia by each color treatment. *N* = 6, except for yellow *N* = 5. Asterisk indicates higher mean compared to the other color treatments (not different among themselves). ANOVA, *P*<0.01.

**Figure 4:**
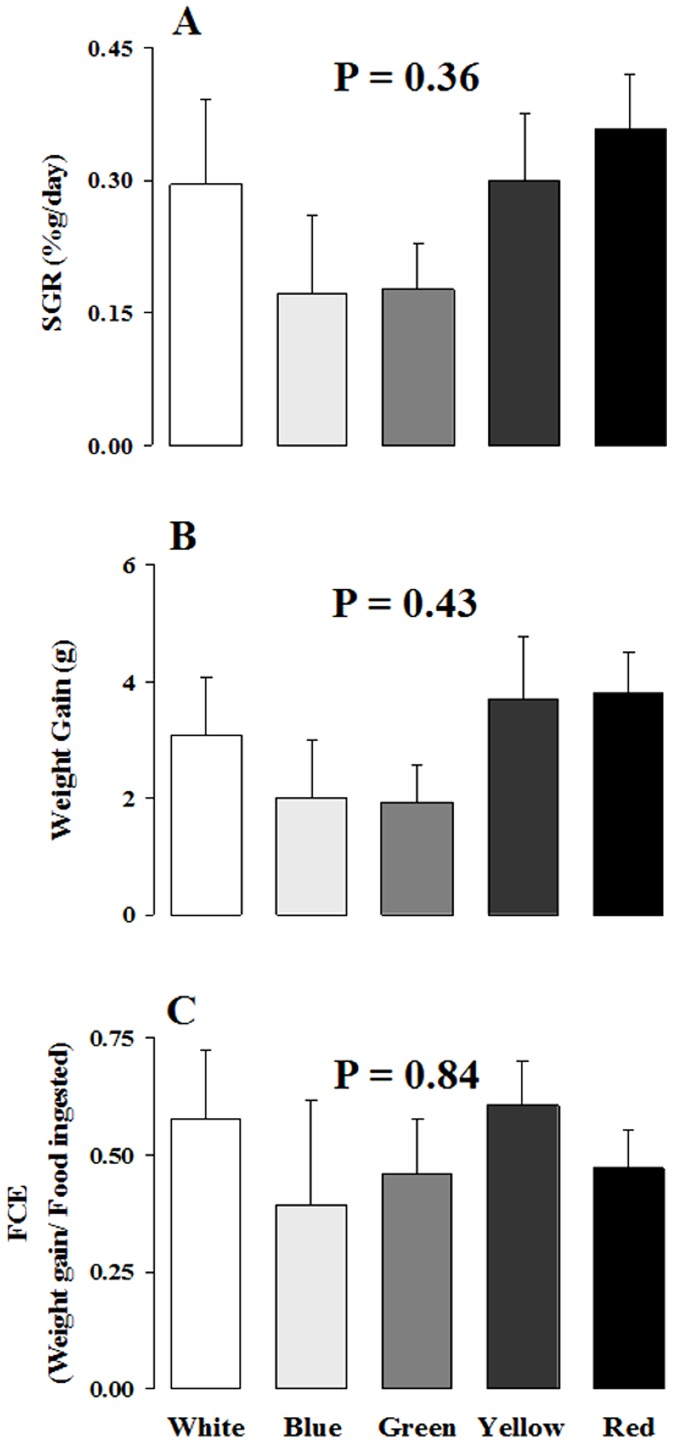
Effects of holding color on growth (A and B) and feed consumption efficiency (C – FCE). Means ± SEM from 6 fish of each color, except for yellow (N = 5). No significant difference was found among the color treatments.

Study 1 revealed that the increased feed intake (Fig. 3) was not converted into a higher growth rate (Fig. 4A, ANOVA, P = 0.36), weight gain (Fig. 4B, ANOVA, P = 0.43) or feed consumption efficiency (Fig. 4C, ANOVA, P = 0.84). High variability in the FCE for each color is not due to errors in collecting uneaten food (all remaining pellets were carefully counted) but is most likely due to the low number of fish analyzed. We also found no significant association between the SGR and the feeding motivation variables (fast feeding and feed intake) for the red light condition [Pearson's correlation: SGR x fast feeding (*r* = 0.71; *P* = 0.11) and SGR x feed intake (*r* = 0.12; *P* = 0.82)].

## Discussion

This study demonstrates that red light color affects the feeding habits of Nile tilapia. Feeding motivation was higher in the red light environment, which resulted in higher feed intake, an effect that has also been reported in humans [20]. This effect occurred even in the absence of visual cues (dark and chemical cues); therefore, the color effect might be stimulating feeding by acting on central control centers. This higher ingestion rate, however, was not converted into higher growth, and thus some disruptive effect of red color might be involved.

Feeding motivation was based on the latency to begin feeding, which was defined as the time taken to start swimming towards food [21] or to the first snatch of food [22]. Here, we found that fast feeding (first snatch to pellets occurring in 10 s or less) was more frequent in fish in red light compared to the other light conditions (Fig. 1). These data supports the hypothesis that red color stimulates motivation for feeding because a lower percentage of fish in the other light colors met the respective time-limit classes.

It is interesting that feeding behavior stimulated by a red environment cannot be attributed to responses exclusively mediated by vision. While fish also ingested pellets in the dark, possibly using chemical cues, test 2 of study 2 clearly showed a chemical effect. Fish were undoubtedly attracted by chemical cues, a fact supporting the hypothesis that red light might stimulate motivation for feeding, thus involving another sensory modality (chemical sense). This discards the theory that response of red-stimulated fish was an effect of vision, but instead is something more deeply in the organism, perhaps in the central nervous system. This is a step stimulating studies on the mechanisms by which light colors might affect animals.

The increased feeding motivation and ingestion in the red light environment reported here has also been described in humans. The color red stimulates appetite in humans, a phenomenon applied in fast food restaurants to increase consumption [20]. Therefore, this effect might be evolutionarily conserved.

During the color treatments, the fish grew as expected considering the feeding regime (once per day, 5 days per week). In the control treatment (white light), the mean SGR averaged 0.3% per day. Higher values (0.4 – 0.6% per day) were reported in this species when fish were fed 7 days a week [6]. These values represent only a fraction of the SGR reported in this species by other authors who used an increased feeding schedule {∼10% of the values reported by [23], who studied ∼12-g fish fed in excess twice a day, or [24], who fed ∼11-g fish four times a day until apparent satiation}. These results are reinforced by the weight gain analyses. The feed intake reported here is a fraction of that reported in non-restricted feeding regimes for Nile tilapia (e.g., feeding to satiation twice per day for 6 days a week [25]). These comparisons indicate that fish in the present study were feed-restricted but were still growing. This condition does not invalidate our conclusions because it was a baseline condition for all treatments. In fact, it reinforces the red color-induced increase in feed intake because fish in all treatments were motivated to feed.

The expected increase in feed ingestion in the red light condition is shown in Fig. 3. This increased feeding, however, was not fully converted into higher growth for these red-acclimated fish (Fig. 4A). Ingested food was converted into a similar level of weight gain irrespective of rearing color (Fig. 4B), thus resulting in similar food conversion efficiencies (Fig. 4C). Red color does not affect weight gain over 4 weeks in individually reared Nile tilapia; instead, red color seems to inhibit growth in this species [6]. Here we showed that red color did not affect the growth of individually reared Nile tilapia over a 4-week following a 4 week acclimation period. The use of an acclimation period reinforced a more pronounced color treatment effect in this study.

As red light stimulated feed ingestion but growth did not increase accordingly, two putative explanations can be drawn: a) the food was not adequately absorbed or b) the absorbed food was channeled to demanding processes (e.g., stress or swimming). Because red light treatment has been shown to change fish metabolism and growth [26], [27] and to increase size heterogeneity in mirror carp [5] and Nile tilapia [6], which suggest negative effects on fish welfare, we consider that the second possibility (metabolized food was channeled to stress or swimming) is more acceptable, but this should be explored in further investigation.
